# Tranexamic acid is associated with decreased transfusion, hospital length of stay, and hospital cost in simultaneous bilateral total knee arthroplasty

**DOI:** 10.17305/bjbms.2020.5060

**Published:** 2021-08

**Authors:** Ryan S. D’Souza, Christopher M. Duncan, Daniel R. Whiting, Michael J. Brown, Matthew A. Warner, Hugh M. Smith, Hilal Maradit Kremers, Thomas M. Stewart

**Affiliations:** 1 Department of Anesthesiology and Perioperative Medicine, Mayo Clinic College of Medicine, Rochester, Minnesota; 2 Department of Orthopedic Surgery, Mayo Clinic College of Medicine, Rochester, Minnesota; 3 Department of Health Sciences Research, Mayo Clinic College of Medicine, Rochester, Minnesota

**Keywords:** Tranaxemic acid, transfusion, total knee arthroplasty, hospital length of stay, healthcare cost

## Abstract

Tranexamic acid (TXA) reduces blood loss and transfusion rates in unilateral total knee arthroplasty (TKA), but there is limited data regarding its efficacy in bilateral TKA. This study reports the impact TXA has on clinical outcomes and hospital cost of care in simultaneous, primary bilateral TKA. The 449 patients were retrospectively reviewed. Primary outcomes included the rates of allogeneic and autologous blood transfusion. Secondary outcomes included hospital length of stay (HLOS), post-hospital discharge disposition, 30-day thromboembolic events (TEE), and mean hospital cost of care. Total direct medical costs were obtained from an institutional research database and adjusted to nationally representative unit costs in 2013 inflation-adjusted dollars. Our study revealed that in patients undergoing simultaneous bilateral TKA, TXA use was associated with reduced allogeneic (OR 0.181, 95% CI 0.090-0.366, *p* < 0.001) and combined allogeneic and autologous transfusion rates (OR 0.451, 95% CI 0.235-0.865, *p* = 0.017). TXA was associated with a HLOS reduction of 0.9 days (β-coefficient −0.582, 95% CI −1.008-−0.156, *p* = 0.008), an increased likelihood of hospital discharge over skilled nursing facility (SNF) (OR 2.25, 95% CI 1.117-4.531, *p* = 0.023) and reduced total hospital cost of care by 6.45% (*p* < 0.001), room and board costs by 11.76% (*p* < 0.001), and transfusion costs by 81.65% (*p* < 0.001). In conclusion, TXA use in bilateral TKA is associated with lower blood transfusion rates, reduced hospital length of stay, reduced cost of hospital care and skilled nursing facility avoidance.

## INTRODUCTION

Numerous trials and meta-analyses have demonstrated that tranexamic acid (TXA) use decreases blood loss, transfusion rate, and cost of care in patients undergoing total knee arthroplasty (TKA) [1-8]. In the fiscal analyses, hospital length of stay (HLOS) appears to be a dominant factor in TKA costs. However, few studies have examined the impact of TXA on the clinical outcomes, HLOS, post-hospital disposition, and total cost of care in patients undergoing bilateral TKA [9-13]. Most investigations on TXA administration in bilateral TKA are of limited sample size and are focused on the clinical efficacy [5,14-18]. Several prospective trials found that TXA decreased the transfusion rates during the bilateral TKA. These results are consistent with the growing body of unilateral joint arthroplasty literature [14-19]. One study observed a reduction in LOS by 0.5 days, a result, which was not statistically significant [18]. Another more recent randomized control trial revealed that the average hospitalization length was reduced by 2.9 days with TXA administration [5]. The goal of the present study was to assess the impact TXA administration has on clinical outcomes, including transfusion practice, hospital length of stay, patient disposition, and financial outcomes in simultaneous bilateral knee arthroplasty.

## MATERIALS AND METHODS

Following institutional review board approval (IRB 10-003336), we retrospectively reviewed all the patients undergoing simultaneous, primary bilateral TKA from 2005 through 2013 in a prospectively maintained institutional total joint registry [20]. The total joint registry provided basic demographic data; additional institutional health care records were carefully reviewed. These included individual medical records, perioperative records, hospital summaries, transfusion documentation, financial records, patient visits, and communications within 90 days after surgery. No patients were lost during this follow-up period.

Inclusion criteria consisted of patients 18 years of age or older undergoing simultaneous primary bilateral TKA between 2005 through 2013. Simultaneous was defined as both knee arthroplasties performed during a single anesthetic. A total of 449 patients were identified. Patients were categorized according to TXA administration status, as identified in Table 1. During the study period, 13 surgeons performed simultaneous, primary bilateral TKA. Two surgeons used TXA routinely in all patients throughout the study period, one surgeon began using TXA in all patients in 2008, and the remaining surgeons did not use TXA prior to 2010. After 2010, TXA administration for TKA became a uniformly adopted practice. Dosing of TXA throughout the study period was standardized, with each patient receiving one gram intravenously (IV) prior to incision of the first knee and one additional gram IV at the time of closure of the second knee. Intraoperative autologous blood salvage was used at the surgeon’s discretion. When used, the blood salvage system was attached to the deep drains in the knees and continued during the patient’s stay. All patients with an autologous salvage volume of at least 120 ml, after preparation, received the salvaged blood regardless of hemoglobin (Hgb) level. No patient received preoperative erythropoietin, and preoperative autologous donation did not occur. All patients received postoperative comprehensive deep vein thrombosis (DVT) prophylaxis, including sequential compressive devices, early mobilization, and chemoprophylaxis using aspirin, low-molecular-weight heparin, or warfarin. Surgical services transfused patients throughout this time period, using a transfusion trigger of Hgb <8 g/dL.

**TABLE 1:**
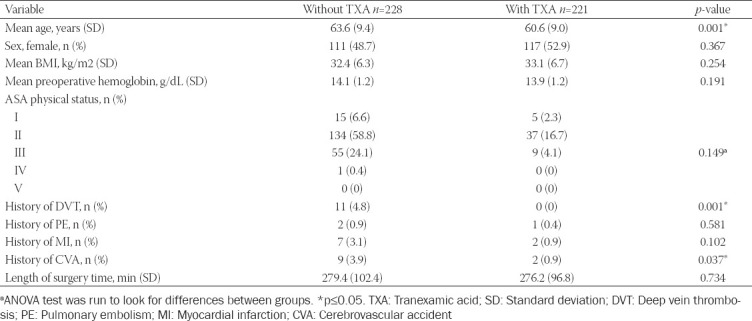
Demographic data based on TXA administration status; SD

Primary outcomes included hospital allogeneic, autologous, and combined allogeneic and autologous transfusion rates. Secondary outcome measures included HLOS, post-hospital discharge disposition, 30-day symptomatic thromboembolic events (TEE) (defined as deep vein thrombosis [DVT] or pulmonary embolism [PE]), 30-day mortality, and direct hospital costs.

An institutional healthcare cost and utilization database was employed to obtain cost data. This database provides line-item details of every procedure or service billed to the TKA patients. Bottom-up micro-costing valuation techniques are employed to generate standardized inflation-adjusted estimates of the costs in constant US dollars [21]. Cost data includes estimates from all payers, but reported costs were limited to direct costs as defined by Medicare Parts A (hospital-billed services) and B (physician services). The gross domestic price implicit price deflator for both Part A and Part B services is used to express the costs for each year in 2013 constant dollars. Mean cost and mean cost differences were calculated for total hospital costs (without the knee implant costs). Total hospital cost included blood utilization, laboratory services, room and board, and pharmacy costs. Our institution prohibits the publication of actual financial cost of care data. Therefore, we report the financial impact of the intervention studied as a percentage cost difference.

Continuous variables were analyzed using student t-tests for differences in means. Proportions were analyzed using the Chi-squared test. We also constructed multiple linear regression models on continuous dependent variables after adjusting for age, gender, preoperative Hgb, American Society of Anesthesiologists’ (ASA) physical status, history of deep venous thrombosis (DVT), history of cerebrovascular accident (CVA), and the year of surgery. For dependent variables with binary categorical outcomes, we conducted binary logistic regression models after adjusting for the aforementioned variables. Given that transfusion practice may significantly vary over time, we also performed a sensitivity analysis to account for changes in allogeneic and autologous blood transfusion rates over the study period. The value of *p* < 0.05 was considered statistically significant. Given the issue of multiple comparisons and the potential for false-positive results, we also adjusted the significance threshold values for each outcome association utilizing the Benjamini–Hochberg false discovery control procedure with a set false discovery rate of 5% [22,23]. All analyses were performed using the SAS version 9.3 (SAS Institute, Cary, NC) and JMP version 10.0.0 (SAS Institute Inc., Cary, NC 27513).

## RESULTS

Demographic data are presented in Table 1. The patient populations were similar with the exception of age, history of DVT, and history of CVA. Patients who received TXA were younger, had no history of DVT, and had a lower incidence of CVA. Clinical outcomes are presented in Table 2. TXA was administered to 221 patients (49%). TXA was associated with a significant reduction in the rate of allogeneic (odds ratio [OR] 0.18, 0.09-0.37, *p* < 0.001) and combined allogeneic and autologous transfusion (OR 0.45, 0.25-0.87, *p* = 0.017). In patients who received an allogeneic transfusion, the number of units transfused was lower in the TXA group versus control (1.74 ± 0.55 vs. 2.56 ± 1.23, *p* < 0.001).

**TABLE 2:**
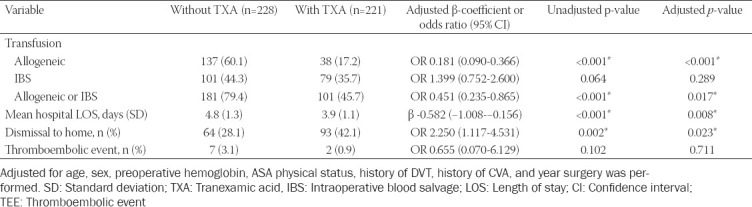
Main outcomes based on TXA administration status; IBS, LOS, CI, and TEE

TXA was associated with a 0.9 day reduction in HLOS (95% confidence interval [CI]: 0.68-1.13, *p* = 0.008). Post-hospital discharge to home vs skilled nursing facility (SNF) was significantly higher when TXA was used (OR 2.25, 1.12-4.53, *p* = 0.023). Significance was maintained in these clinical outcome measures after adjustment for age, preoperative Hgb, ASA physical status, history of DVT, CVA, and year of surgery. Allogeneic transfusion was associated with an increased HLOS of 0.8 days (95% CI: 0.52-0.99, *p* < 0.001) irrespective of TXA use. Similarly, allogeneic transfusion was associated with a 2.5 times greater requirement for SNF admission (45% vs. 19%, *p* < 0.001). TXA use was not associated with an increase in 30-day TEE. No patients died within 30 days of surgery.

Hospital cost data are presented in Table 3. Compared to the control cohort without TXA, the administration of TXA was associated with a reduction in mean total direct hospital cost (excluding implant costs) by 6.45% (95% CI: 1.93-10.95%, *p* < 0.001). Furthermore, TXA administration was also associated with a reduction in room and board costs by 11.76% (3.94-19.59%, *p* < 0.001), transfusion costs by 81.65% (66.97-95.87%, *p* < 0.001), and laboratory costs by 55.40% (44.46-66.04%, *p* < 0.001). TXA administration was associated with higher pharmacy costs by 29.77% (22.64-36.99%, *p* < 0.001).

**TABLE 3:**
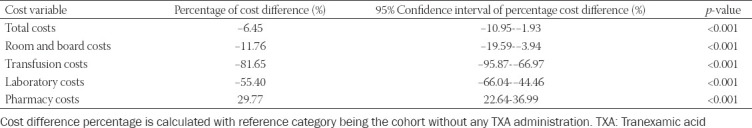
Hospital cost data based on TXA administration status

After adjustment for multiple comparisons using the Benjamini–Hochberg false discovery control procedure, all statistically significant associations remained significant. Sensitivity analysis also revealed no significant differences in allogeneic transfusion rates (*p* = 0.119) and autologous transfusion rates (*p* = 0.613) over the study period in our cohort.

## DISCUSSION

The primary goal of this study was to investigate differences in allogeneic and autologous transfusion rates in patients undergoing simultaneous bilateral knee arthroplasty with and without TXA administration. The use of a standardized TXA dosing regimen resulted in a significant reduction in allogeneic and autologous transfusion requirements. Our data are consistent with prior published studies with smaller patient populations [13,14,18,24,25]. Our transfusion rate was greater in both groups compared to the trial by Kim et al., which reported transfusion rates of 20% without TXA compared to 5% with TXA [16]. This difference in transfusion rate is likely a result of a strict transfusion trigger of <7 g/dl in the Kim et al. study compared to a more liberal transfusion threshold of Hgb < 8 g/dl in our cohort.

In addition to investigating the effects of TXA on transfusion, this study also evaluated the effects of TXA on multiple secondary outcomes. In unilateral TKA, both TXA and transfusion avoidance have been associated with a reduction in HLOS, reduced estimated direct hospital costs, and a discharge disposition favoring home versus SNF [5,11,12,26-28]. Our study indicates that TXA is associated with similar benefits as in the bilateral TKA patient population. This study similarly reports a significant reduction in LOS with a mean decrease of 0.9 days in TXA patients. Karam et al. compared LOS in patients undergoing simultaneous bilateral TKA with and without TXA but did not find a statistically significant difference in length of stay [18]. However, a more recent randomized control trial revealed that the average hospitalization length was reduced by 2.9 days with TXA administration [5]. Transfusion is an independent risk factor for increased HLOS. The increased LOS reported herein is consistent with the results reported in two recent studies analyzing nationwide inpatient data from 2000 to 2009, showing an increase HLOS associated with transfusion after TKA [11,27].

Unlike other studies of simultaneous bilateral TKA, this study investigated the estimated mean direct hospital costs between patients who did and did not receive TXA. We report a significant cost reduction in mean total direct hospital cost when TXA is used. The financial savings are the result of reductions in hospital room and board, laboratory, and blood utilization costs, despite the increase in pharmacy costs associated with TXA administration. Although no other studies have investigated the relationship of cost with TXA in simultaneous bilateral TKA, it has been studied in unilateral TKA with similar results. According to Gillette et al., unilateral TKA with TXA reduces total direct hospital costs by $879 (5.5% cost reduction), despite the increased pharmacy cost associated with TXA [10]. This association is also concordant with another cost-benefit analysis study in the lumbar spine surgery population that reported a $328.69 cost saving per patient in the TXA group undergoing long-length spinal constructs [6].

Our study also examined hospital dismissal disposition in relation to allogeneic transfusion and TXA administration for bilateral TKA. Allogenic transfusion was associated with an increased likelihood of requiring SNF care. TXA was associated with SNF avoidance. In adjusted analyses, patients who received TXA were 2.25 time more likely to be dismissed directly to home. One could assume that the overall cost of care savings would be significantly higher had we been able to measure the costs associated with SNF admission and SNF LOS.

At this time, there are no other known, published studies of simultaneous, primary bilateral TKA and the association between TXA, transfusion, and disposition after hospital discharge. Research from unilateral total joint arthroplasties (TJA) has shown a significant increase in discharge to home with TXA, as well as a significant correlation between receiving an allogenic transfusion and discharge to a SNF [11,20,27]. While there are many factors that may influence whether a patient is discharged to home or SNF, these results indicate that TXA may also help avoid the additional costs associated with discharge to a nursing facility. Other strengths of our study include the utilization of a multivariable regression analysis that controls for potential confounding factors and reduces the likelihood for information bias. We also performed adjustment for multiple comparisons using the Benjamini–Hochberg control procedure to reduce the risk for type I statistical error. Finally, given the concern for changes in transfusion practice over time, we performed a sensitivity analysis that demonstrated no significant difference in transfusion rates over the study period.

Potential weaknesses of this study are those associated with the inherent limitations of retrospective study design. This includes the potential for bias related to allogeneic transfusion thresholds, patient selection for TXA administration, and failure to capture thromboembolic events in the medical record. It is challenging to determine casual relationships within this observational study design. Differences in the patient population, changes in transfusion practice, and hospital efficiency during a long study period spanning 2005-2013 may play a role. Additionally, we defined TXA administration as a binary variable: with or without TXA. We did not abstract data on TXA dosing and it would be interesting to determine if there is a dose-response relation between TXA dose and transfusion rates. Certainly, other unmeasured factors other than the use of TXA or transfusion that was not discovered during our review of the medical record could have affected LOS. However, given the large number of patients included in our study, we were able to demonstrate clinically significant relationships between LOS, TXA, and transfusion rates after multivariable adjustment for important baseline demographic and clinical variables. Furthermore, while we abstracted data on major comorbidities such as myocardial infarction (MI), CVA, DVT, and PE, we did not abstract extensive data on other comorbidities that would have helped us determine a comprehensive comorbidity score, such as the Charlson comorbidity index. We also did not abstract other pertinent data on tourniquet use and intraoperative fluid administration. Also, specific decision to administer TXA to individual patients reflects not only the surgeon preferences but consensus about drug safety by anesthesia care providers. Finally, it is possible that a certain percentage of patients who returned home for follow-up care may have experienced a TEE which was not reported in the institutional medical record. However, the standard of care throughout the study period is to have patients return to the orthopedic clinic 2–3 months after the surgery. At this time, any reported medical or surgical complications are entered into the institutional joint registry, decreasing the likelihood of missing such events.

## CONCLUSION

TXA use in bilateral total knee arthroplasty is safe and associated with lower blood transfusion rates, reduced hospital length of stay, reduced total cost of hospital care, and skilled nursing facility avoidance. TXA may be considered in patients undergoing bilateral total knee arthroplasty who do not have a preexisting contraindication to the drug.
